# Response to Thomsen et al.: Comments on “The Radiological Physics Center's standard dataset for small field size output factors”

**DOI:** 10.1120/jacmp.v15i2.4841

**Published:** 2014-03-06

**Authors:** David S. Followill, Stephen Kry

**Affiliations:** ^1^ Radiological Physics Center U. T. M. D. Anderson Cancer Center Houston TX USA

Dear Editor,

The authors of “The Radiological Physics Center's standard dataset for small field size output factors” wish to respond to the comments by Thomsen et al. After an error due to the inclusion of incorrect data in the Varian data was noted, the authors submitted an Erratum to the *Journal of Applied Clinical Medical Physics.* Thomsen and colleagues were made aware of the Erratum and the new data. The authors and RPC staff stand behind the data presented in the original manuscript and the corrected Varian data in the Erratum. While the Erratum data are different (maximum difference of 2.9%) and warranted new values, we do not believe they represent a “major” change, as claimed by Thomsen et al.

The RPC's data were generated from numerous onsite dosimetry review visits to radiation oncology facilities participating in the National Cancer Institute's (NCI)‐funded clinical trials. These data represent, for Varian beams, between 22 and 136 measured datasets. Thomsen and colleagues present a combination of measured and treatment planning system (TPS) calculated data from only four facilities using three different dosimetry systems (one of which was a prototype). Thomsen et al. also give very little information as to their measurement setup. I have assumed that they followed the measurement setup in the original manuscript, but this is only an assumption. More detail on how the measurements were made would have been helpful in order to be able to perform a direct comparison of their data and that from the RPC.

The output factor data tables presented by the RPC clearly distinguish between the RPC measurements and the TPS calculations performed by the many visited radiation oncology facilities. Thomsen et al. have made the mistake of labeling data in their graphs as “RPC Calculated” data when, in fact, those data were calculated by the many institutions. Thomsen et al.'s figures show a mixture of measured and TPS calculated data from their four facilities. However, no distinction is made in their analysis between a measured value and that which is calculated from a TPS beam model, which is warranted given the difference noticed in the extensive evaluations by the RPC. The figures also do not clearly illustrate which data points are measurements and which are calculations, but show instead a large spread in the data. The medical physicists from the Radiological Physics Center (RPC) use an identical measurement procedure, irradiation setup, ion chamber (Exradin A16, $\text{volume}=0.007\;{\text{cm}}^{3}$), and electrometer for all of the measurements. This is noted in the paper as one of the reasons for the very small standard deviation of the RPC measurements and associated output factors.

Thomsen et al. differentiated between TrueBeam machines and older Varian models. The RPC now has enough data for the Varian TrueBeam machines (n>5) for the 6 and 15‐16 MV beams to generate standard data based on our measurements. In Table 1, the TrueBeam data are presented in bold italics beneath the RPC standard values (from the Erratum) for all Varian linacs. Except for the smaller field sizes for the 15‐16 MV beams, there is a negligible difference between the Erratum data and the subset of TrueBeam data. To say the TrueBeam data are different or the same as from the other Varian machine data is not appropriate yet, until we have more results. The small amount of data listed above suggest that it is similar. The TrueBeam flattening filter‐free (FFF) beam data do show differences, as we would expect. I assumed that Thomsen et al.'s data were from the regular TrueBeam photon energies and not the FFF beams; however, this was not stated in their comment.

**Table 1:** The RPC‐measured and institution treatment planning system‐calculated small field size dependence output factor values for Varian machines. The values in square brackets and parentheses beneath each energy for each field size value are the average absolute percent differences and standard deviations of the values, respectively. For each energy and field size, the number of measurements (accelerators) is also shown. Values in italicized parentheses indicate RPC measured values for Varian TrueBeam

*Field Size (cm)*	*Varian 6 MV RPC Institution*		*Varian 10 MV RPC Institution*		*Varian 15 MV RPC Institution*		*Varian 18 MV RPC Institution*	
10×10	1.000	1.000	1.000	1.000	1.000	1.000	1.000	1.000
6×6	**0.938**	0.945	**0.959**	0.964	**0.963**	0.964	**0.969**	0.971
	(0.937)*				*(0.965)*			
	(0.005)	(0.008)	(0.002)	(0.003)	(0.005)	(0.007)	(0.003)	(0.006)
	[0.8%]		[0.5%]		[0.4%]		[0.5%]	
	(n=127)		(n=22)		(n=32)		(n=41)	
4 X 4	**0.886**	0.9 00	**0.916**	0.9 27	**0.927**	0.928	**0.928**	0.933
	*(0.885)*				*(0.924)*			
	(0.006)	(0.013)	(0.006)	(0.010)	(0.006)	(0.009)	(0.002)	(0.009)
	[1.8%]		[1.2%]		[0.7%]		[0.9%]	
	(n=122)		(n=25)		(n=32)		(n=37)	
3×3	**0.851**	0.8 70	**0.880**	0.894	**0.892**	0.894	**0.884**	0.891
	*(0.849)*				*(0.884)*			
	(0.007)	(0.012)	(0.006)	(0.008)	(0.006)	(0.012)	(0.005)	(0.019)
	[2.4%]		[1.6%]		[0.9%]		[1.4%]	
	(n=123)		(n=25)		(n=31)		(n=41)	
2×2	**0.804 *(0.802)***	0.8 25	**0.823**	0.8 38	**0.824 *(0.814)***	0.823	**0.806**	0.804
	*(0.802)*				*(0.814)*			
	(0.008)	(0.020)	(0.005)	(0.015)	(0.011)	(0.040)	(0.007)	(0.020)
	[2.9%]		[2.0%]		[2.2%]		[1.6%]	
	(n=136)		(n=23)		(n=33)		(n=40)	

We find it interesting that the Thomsen et al. data presented have a large standard deviation, and have mean output factors for each field size that are comparable to our manuscript's Institutional TPS‐calculated data. It would have been more informative to see Thomsen et al.'s measured and calculated data separated out in order to better understand their agreement or disagreement with the RPC measured data. The biggest difference between the RPC measured data and the Thomsen data occurs for the 6 MV photon beam, where the majority of the Thomsen data are TPS calculations, which happen to agree very well with the Institution TPS‐calculated values presented in the Erratum and shown in the Table. This is where the RPC, as well as Kron et al.,^(1)^ has shown there to be issues with beam modeling that hinder the calculation of the correct output under these small field conditions. The difference between the RPC Erratum Varian data and the Thomsen data for the 10 and 15 MV beams (Thomsen data are mainly measured data for these two beam energies) is very small and agrees within measurement uncertainty.

Finally, the RPC data are to be used as a quality assurance tool and not to replace the need to perform one's own measurements and correctly model their photon beams. I believe Thomsen et al. overstated their conclusion somewhat by stating “great caution must be advised if using the RPC dataset as a reference …”. All medical physicists must use their best judgment as educated individuals to understand when data may not agree, and to try and seek answers as to why. Clearly, the RPC data have accomplished its task with Thomsen et al. since they performed the analysis shown in their comment. However, did Thomsen and colleagues scrutinize their own data enough to fully understand why they differed from a very large reproducible measurement dataset, or did they assume their own data were correct and without question?
